# Presentation of Patient Sixteen Months after Modified Kraske’s Operation for Extirpation of Rectum1Read at the thirty-second annual meeting of the Medical Association of Central New York, at Syracuse, October 17, 1899.

**Published:** 1900-02

**Authors:** S. L. Elsner

**Affiliations:** Rochester, N. Y.


### PRESENTATION OF PATIENT SIXTEEN MONTHS AFTER MODIFIED KRASKE'S OPERATION FOR EXTIRPATION OF RECTUM.[1]

By S. L. ELSNER, M. D., Rochester, N. Y.

THIS patient, aged 60, a farmer, residing in Honeoye Falls, N. Y., first consulted me in May of last year. His family history is good. For a short time, some eighteen years ago, he suffered from hemorrhoids, otherwise he had always enjoyed good health. About ten months before seeing me he began to have constipation and during evacuations felt a burning pain in rectum. His stools soon contained blood, pus and mucus and were very offensive. When his bowels were not loose there was evidence of a stricture by the form of the stool. Pain steadily grew worse, bleeding more frequent and the discharges more offensive. Great loss of strength and flesh followed. Examination at this time revealed that he was poorly nourished. Weight, 136 lbs; markedly cachectic. His heart, lungs, liver and spleen normal; pulse rapid and temperature slightly elevated. Urine, 1025, loaded with phosphates, but otherwise negative. The rectum showed a carcinoma, beginning at the sphincter and taking in the left half of the rectal wall for about one inch. The second inch and a half of the gut was a complete circle of cancer with a marked stricture at its upper extremity. On asking the patient to make pressure this circular part of the growth came forward and I could feel healthy mucous membrane above it.

He had up to this time been treated for "hemorrhoids," "ulcerated rectum," and had frequently been dilated with the rectal bougie. In fact, one of our advertising quacks, after looking at his tongue and feeling of his pulse, was ready to guarantee a cure with three treatments.

After spending one month more in arranging his business affairs and getting the advice of some reputable surgeons, he returned to

1. Read at the thirty-second annual meeting of the Medical Association of Central New York, at Syracuse, October 17, 1899.

Rochester in June, 1898, prepared to chance a radical operation. Examination at this time showed the growth to have increased, involving now considerable of the anterior wall over the prostate. It was also with difficulty that I could reach above the stricture. He was suffering more pain; there was more obstruction, and it required a great effort for him to urinate. He entered the hospital and during the first four days received thorough preparatory treatment. This consisted of the ingestion of a large amount of fluids and copious and frequent flushings of the colon. Operation, June 25, 1898. A left iliac colostomy was completed, all but the opening in the gut. The patient was then placed in an exaggerated Sims's position. An incision, from the left of the second sacral spine, curving toward the median line and reaching the anus, was made. The soft parts were retracted and thus the lower left half of the sacrum and coccyx were exposed. The left sacro-sciatic ligament was divided. After removing the coccyx, the posterior wall of the diseased gut presented. The posterior attachments of the rectum were now loosened from the anterior surface of the sacrum and the fifth and lower half of the fourth vertebra of that bone excised. The upper extremity of this section ended one third inch below the third sacral foramen.

Thinking that it would be possible to excise well beyond the diseased portion of gut and yet not open the peritoneal cavity, I proceeded to free the rectum. On reaching the lower portion my fingers broke through into the lumen of the gut in several places. At this stage in the operation I could definitely appreciate the extent of the prostatic involvement, and the utter impossibility of saving even the muscles. An artificial anus in the sacral region was most impracticable and so I decided to modify the operation and close up the proximal end of the gut. This also avoided opening the peritoneum. The section of the rectum was made opposite the edge of the cut sacrum and about threefourths of an inch above diseased tissue. After irrigating the parts, the lower wound and growth were temporarily packed with gauze. The gut was closed with two rows of sutures and fastened behind the sacrum. The diseased rectum with the portion of the prostate involved were then rapidly excised. Hemorrhage was insignificant. In the deep parts of the wound a few stay sutures were introduced and tied. The skin was sutured in part, leaving room above and below for a large gauze packing. Anesthesia was chloroform. The time occupied two and a half hours.

The patient reacted well; at the end of twenty-four hours a small opening was made in the colon. There was no distension and his highest temperature was 100° F. The nausea soon ceased and during the second twenty-four hours he was able to retain fluids. On the second day the opening in the colon was enlarged to the extent of the outer wound. A temporary silk ligature was inserted on each side of the intestinal wall. Making traction on these so pulled up the posterior wall of the gut, that it shut off any direct communication between the gut below and above the opening. These strands of silk were fastened to the adjoining skin on either side and insured against anything passing into the blind pouch. On the sixth day the patient was taking solid food, and he had one or two stools daily.

In making this colostomy the three layers of muscle were separated, each in the direction of its fibers, so that now he has considerable control of the passage and his movements are involuntarily only when by chance his bowels are loose.

The wound in the back was packed and irrigated daily. He sat up in bed during the seventeenth day. On the twentieth day he was out of bed. He walked about during the fourth week and left the hospital for his home six weeks after operation. His weight when discharged was 149 pounds; gain, twelve pounds. For the further treatment of the wound, I am indebted to Dr. H. S. Benham, of Honeoye Falls. In February last, eight months after operation, he had gained about forty pounds and stated that he never enjoyed better health.

R. Belin, in speaking of the iliac colostomy in the radical cure of rectal cancer, October, 1899, states that only a single case is recorded where Kraske's operation was immediately performed after a colostomy. This will be another added to the list, and, I believe, the first in which the end of the gut was successfully closed.

This case demonstrates what radical measures, at times, accomplish for apparently hopeless conditions, and further that colostomy should not be a *dernier* resort since it is a valuable therapeutic measure. It antagonises recurrence and prevents infection at the point of excision.

83 St. Paul St.

---

Incontinence of Urine.—Lycopodium has been used with success for this affection in children. Twenty drops of the tincture should be given three times a day and this may be increased to forty or fifty drops. It is, in some cases, more efficient than belladonna.

